# Molecular and virulence differences of *Klebsiella pneumoniae* isolated from blood

**DOI:** 10.3389/fmicb.2025.1650010

**Published:** 2025-07-30

**Authors:** Zhaoxia Xu, Yuxuan Xiong, Xueguang Duan, Jing Han, Xing Xiang, Ran Han, Shengwei Zhang

**Affiliations:** ^1^Department of Clinical Laboratory, Dongfang Hospital, Beijing University of Chinese Medicine, Beijing, China; ^2^Beijing University of Chinese Medicine, Beijing, China

**Keywords:** *Klebsiella pneumoniae*, BSIs, molecular, virulence, CRKp, hvKp

## Abstract

**Background:**

Bloodstream infections (BSIs) accompanied by sepsis with *Klebsiella pneumoniae* (*K. pneumoniae*) represents a public health threat being potentially life-threatening. There have been an increasing number of reports on *K. pneumoniae* isolates in China. We conducted a case-based genomic and experimental study. We studied the diversity of *K. pneumoniae* isolated from blood causing sepsis to reveal differences between patients.

**Methods:**

The isolates from six patients infected with *K. pneumoniae* from January 2022 to April 2023 were analyzed by antimicrobial susceptibility testing and sequenced by whole genome sequencing (WGS). The data collected were used to investigate their serotype, molecular subtype, and virulence-associated and antimicrobial resistance (AMR) genes contents as well as the presence of plasmids.

**Results:**

WGS data revealed that six isolates clustered in 5 different genetic types, 3 of which identified as carbapenem-resistant *K. pneumoniae* (CRKp) isolates, 2 as hypervirulent *K. pneumoniae* (hvKp) isolates. Among them, the serotype of Kpn3 is ST950, which is a relatively new serotype strain in China. CRKp isolates were resistant to almost all antibiotics and carries multiple plasmids with different resistance genes. They all contained the KPC-2 gene, but their *bla*_KPC-2_-harbored plasmids were different. 2 hvKp isolates belonged to 2 different sequence types, ST23 and ST65, respectively. HvKp with a hypermucoviscosity phenotype had a higher mortality rate in mice. However, they had less plasmid and antimicrobial resistance genes than CRKp, and were susceptible to all tested antimicrobial drugs.

**Conclusion:**

This study provided important insights into the diversity between *K. pneumoniae* strains isolated from blood in the same hospital. *K. pneumoniae* isolated from different patients has diversity of drug resistance genes, virulence genes and plasmids, which may affect the outcome of patients. Therefore, accurate treatment of patients according to the molecular characteristics and drug resistance phenotype of the isolates will achieve better efficacy.

## 1 Introduction

*K. pneumoniae*, a Gram-negative opportunistic pathogen that is responsible for severe infection resulting in sepsis, such as urinary tract infections, pneumonia, liver abscesses, bloodstream infections (BSI) (Choby et al., 2020; Guerra et al., 2022; Martin and Bachman, 2018; Bengoechea and Sa, 2019). Newborns, the elderly, and immunocompromised individuals are at high risk of *K. pneumoniae* infection (Bengoechea and Sa, 2019). *K. pneumoniae* is the second leading cause of BSI caused by Gram-negative bacteria, with a mortality rate of ~20–30% (Martin and Bachman, 2018). Classical *K. pneumonia* strains are intrinsically resistant to ampicillin, carbenicillin and ticarcillin through the production of a chromosomal penicillinase (SHV-1) (Dai and Hu, 2022). Recently, it is increasingly detected a multidrug-resistance due to the acquisition of different antibiotic resistance genes carried on the chromosome or mobile elements (plasmids and transposons) (Stojowska-Swędrzyńska et al., 2021). This reason led to a more challenging treatment of multidrug-resistant (MDR) *K. pneumoniae* clones (Moradigaravand et al., 2017). In particular, a growing number of carbapenem—resistance *K. pneumoniae* strains has emerged in recent years due to the extensive use of carbapenems in clinical practice (Chang et al., 2021). Carbapenem-resistant *K. pneumoniae* (CRKp) refers to *K. pneumoniae* that have developed resistance to Carbapenem antibiotics such as imipenem, meropenem, and ertapenem. They generally carry carbapenemase genes (such as *KPC*, *NDM*, *OXA-48*, *VIM*, *IMP*, etc.). CRKp isolates were the most urgent priority bacteria and urgent threat by the World Health Organization (WHO) and US Centers for Disease and Control (CDC) (Hu et al., 2020). The main mechanism of carbapenem resistance is mediated by the accessory genome. The plasmid-mediated carbapenemases mechanism represents the one of major concern (Martin and Bachman, 2018). The most commonly detected carbapenemases of *K. pneumoniae* (KPC) is the *β*-lactamases (Martin and Bachman, 2018; Vubil et al., 2017). KPC-2 represents the most common variant of KPC enzymes, contributing to the resistance to all *β*-lactam antibiotics (Chen et al., 2022; Tang et al., 2022). The transmission of KPC genes is mediated through different mechanisms including mobility of small genetic elements, horizontal plasmids transfer and clonal spread (Chen et al., 2014). Besides KPC gene, New Delhi metallo-β-lactamase-1 (NDM-1), Verona-integron encoded metallo-β-lactamase carbapenemases (VIM), Imipenemase (IMP) type class B metallo-β-lactamases (MBLs), Imipenemase (IMP) type MBLs, and OXA carbapenemases are involved in the carbapenem resistance as well (Martin and Bachman, 2018; Paczosa and Mecsas, 2016; Hobson et al., 2022). Hypervirulent *K. pneumoniae* (hvKp) strains, which are characterized by a hypermucoviscosity phenotype, may be responsible for several community-acquired invasive and life-threatening infections with devastating consequences (Mukherjee et al., 2021; Hua et al., 2022). The virulence factors (VFs) that determine the severity of the infections include capsule, lipopolysaccharide, siderophores, pili, allantoin utilization, other iron uptake systems, efflux pumps, and a type VI secretion system (Martin and Bachman, 2018; Clegg and Murphy, 2016; Remya et al., 2019). The *rmpA*, *rmpA2*, *aerobactin* genes, positive string test were features of the hvKp (Wang et al., 2020). The main serotypes of hvKp were K1/K2/K5/K20/K54/K57 (Wang et al., 2020; Zhou et al., 2023).

At present, the commonly used clinical methods for the identification of *K. pneumoniae* are automatic microbial identification system and MALDI-TOF MS. These two methods can only identify colony species. This is not enough for precision therapy. Because differences in bacterial genomes lead to differences in bacterial virulence and drug resistance, there will be different treatment effects for patients infected with the same pathogen. Whole genome sequencing (WGS) can comprehensively analyze the source, drug resistance and virulence of bacteria, which provides great help for patient precision treatment.

Differences in phenotypic and molecular characteristics of *K. pneumoniae* affect its pathogenicity, which may ultimately affect the prognosis of patients. The diversity of *K. pneumoniae* genomes can lead to differences in drug resistance and virulence, which may affect patients’ different responses to empiric medication and outcomes. Therefore, timely and accurate identification of *K. pneumoniae* types, drug resistance genes, virulence factors and plasmids are helpful for clinicians to rationally select antibiotics and adjust treatment strategies (Raffelsberger et al., 2021). At present, there are few studies on the diversity of *K. pneumoniae* in the same hospital or region.

Aim of this study was to investigate the molecular characteristics by WGS and phenotypes of *K. pneumoniae* strains isolated from patients with sepsis. Provide the basis for the clinical precise treatment of patients.

## 2 Methods and materials

### 2.1 Patient enrollment and sampling

A cohort study was performed on *K. pneumoniae* infections diagnosed in BSI cases with sepsis at a tertiary hospital of Dongfang Hospital, Beijing University of Chinese Medicine located in Beijing, China. The clinical history of the patients admitted to the hospital from January 2022 to April 2023 were collected from the records. It mainly includes clinical symptoms, invasive procedures performed, therapeutic use of antimicrobial drugs, and outcomes in the 30 days following the diagnosis of *K. pneumoniae* infection. The patients’ ages ranged from 62 to 91 years old (with an average of 79.5 years old), including 2 males and 4 females. All cases were from inpatients and met the criteria of Sepsis 3.0. A written informed consent was provided by the patient or by the patient’ next of kin. The study was approved by institutional review board (IRB) of Dongfang Hospital Beijing University of Chinese Medicine with a reference number of JDF-IRB-2022000112.

Patients with *K. pneumonia* bloodstream infection sepsis meet the following criteria: 1. Blood culture positive for *K. pneumonia*; 2. Third International Consensus Definitions of Sepsis and Septic Shock (Sepsis 3.0).

### 2.2 Blood culture and bacterial identification

A total of 20 mL of venous blood from the patient’s upper limb was collected and injected into both aerobic and anaerobic culture bottles of 10 mL. Culture bottles were placed on the blood culture instrument that gave an alarm once the bacteria grew. Then, 2–3 drops of positive culture medium were inoculated onto blood agar plates and Chinese blue agar plates at 35°C, with 5% of CO_2_ for 48 h. Several growing colonies were identified using MALDI-TOF MS (Bio Mérieux, Lyon, France) and isolates were designated as Kpn1, Kpn2, Kpn3, Kpn4, Kpn5 and Kpn6.

### 2.3 Antimicrobial susceptibility testing

Antimicrobial susceptibility testing (AST) was performed using the VITEK-2 Compact system (bioMérieux, France) by the photoelectric turbidimetry for piperacillin-tazobactam, ceftazidime, ceftriaxone, cefepime, ertapenem, imipenem, amikacin, levofloxacin, sulfamethoxazole, and cefperazone-sulbactam. The susceptibility to tigecycline was determined by the broth dilution method. The minimum inhibition concentrations (MIC) were interpreted for all the antibiotics according to the M100 manual of the Clinical and Laboratory Standards Institute (CLSI) guidelines (CLSI, 2022), while MIC values for tigecycline were assessed according to the European Committee on Antimicrobial Susceptibility Testing (EUCAST) criteria.^1^

### 2.4 Whole-genome sequencing (WGS)

Genomic DNA from the isolates was extracted using IndiSpin Pathogen Kit (Indical Bioscience, Stockholm, Sweden) and DNA concentrations was measured with the Qubit dsDNA HS (High Sensitivity) assay Kit (Thermo Fisher Scientific, Massachusetts, United States). Genomic DNA libraries were constructed using the NEBNext Ultra II DNA Library Prep Kit for Illumina (NEB, Massachusetts, United States) and using the ligation library preparation kit SQK-LSK109 (Oxford Nanopore Technologies, Oxford, UK) for the Oxford Nanopore systems. The libraries were sequenced by the NovaSeq 6,000 platform (PE150, Illumina, California, United States) and by the MinION platform (Oxford Nanopore Technologies, Oxford, UK) using R9.4.1 flow cells (FLO-MIN106). The quality of the raw sequence reads was assessed for both the platforms, and low-quality reads, adaptor contaminations, duplications, and short reads were removed to facilitate the assembly.

### 2.5 Genome assembly and bioinformatics analysis

A combination of long-reads (Nanopore MinION) and short-reads (Illumina NovaSeq 6,000)—based platforms was used to assemble the complete genome sequences of the strains. This hybrid *de novo* assembly was performed using Unicycler v0.4.7 refering to this article (Wick et al., 2017). The highly accurate Illumina reads were mapped against the MinION reads that served as a reference genome to correct any random sequencing errors and to obtain an assemble of high quality. Multilocus sequence type (MLST), antimicrobial resistance (AMR) genes, and plasmid replicons were investigated using the following databases: MLST 2.0 (with the min. Depth for an allele of 5×), ResFinder 4.1 (with threshold 90% and minimum length 80% for both chromosomal point mutations and acquired antimicrobial resistance genes), and PlasmidFinder2.1 (with cutoff values of 90% identity and 80% of minimum coverage) respectively^2^ (Martins et al., 2020), while the presence of VF was assessed using the Virulence Factor database^3^ (Jia et al., 2023). The identification of capsular types within the assembled sequences was determined by Kaptive software^4^ (Ahmed et al., 2021). Upload the Fastq file to Proksee, select Flye for assembly, with a coverage depth of ≥30×, and click “Assemble.” The assembled sequence is loaded into the visualization interface, and gene tags (such as drug resistance gene *KPC-2*) are manually added or modified. Export high-definition images. The plasmid genome circle maps were visualized using proksee^5^ (Ni et al., 2022).

### 2.6 *wzi* gene sequencing

Primers targeting the *wzi* gene were designed: for *wzi*_F (5′- GTGCCG CGA GCG CTT TCT ATC TTG GTA TTC C-3′) and *wzi*_R (5’-GAGAGC CAC TGG TTC CAG AA[C or T] TT[C or G] ACC GC-3′) according to Brisse et al. (2013). Sanger sequencing was performed from the 580-bp DNA fragments obtained on both strands using the PCR primers. The sequences obtained were compared to the *wzi* alleles data obtained from Brisse et al. (2013) and predict the corresponding capsular (K) types between the strains.

### 2.7 Phylogenetic analysis

CRKp clones recently described in Europe (ST11, ST15, ST101, and ST258), hvKp clones of ST23 and ST65 and the NTUH-K2044 reference strain (accession number: NC_012731.1) were aligned using ClustalW2 for phylogenetic analysis (Larkin et al., 2007). Maximum Likelihood (ML) phylogenetic tree was generated using IQ-TREE with 1,000 bootstrap replicates (Wyres KA-O et al., 2020; Wyres K. L. et al., 2020; Wyres et al., 2019).

### 2.8 Mucoviscosity

CRKp strains were cultured overnight in Luria-Bertani liquid medium at 37°C and mucoviscosity was assessed by low-velocity centrifugation. The cultures were normalized to OD_600_ of 1 at 22°C and centrifuged at 100 g for 20 min (Marathon 3000R; Fisher Science). Determining the turbidity of supernatant at OD_600_ (BioMate 3 Thermo Spectronic; Fisher Scientific). Each sample was measured three times (Cheng et al., 2022).

### 2.9 CPS quantitation

CPS was extracted and the concentration of uronic acid was quantified according to the established method (nanomoles per 10^9^ CFU). Each sample was measured three times (Favre-Bonte et al., 1999).

### 2.10 Mice infections

Male ICR CD1 mice (Harlan) weighing 20 to 25 g were immunosuppressed by two doses of cyclophosphamide (150 mg/kg 4 days and 100 mg/kg 1 day prior to infection) and two doses of cortisone subcutaneously (20 mg/kg 4 days and 1 day prior to infection). 10 μL overnight cultured *K. pneumoniae* were inoculated into Luria-Bertani liquid medium. Incubate at 37°Cfor 3 to 5 h until the logarithmic phase (OD600 = 0.6). Adjust the concentration of the bacterial to 1×10^6^ CFU/mL using 1 mL saline. In the mortality study, mice (10/group) were infected by lateral caudal vein with 100 μL bacterial solution. The infected mice were placed in cages and raised normally. Observe the survival status once a day. The mice were followed up until death or for 10 days (Cheng et al., 2022).

### 2.11 Statistical analysis

Quantitative data are expressed as means and standard errors. Mann–Whitney U test was used for statistical comparisons of 2 groups. Kruskal-Wallis test was used for statistical comparisons of > 2 groups. Survival curves were calculated according to the Kaplan–Meier method and compared using log-rank test. For all analysis, *p*-value < 0.05 was considered significant. All statistical analysis in this study used Graphpad prism software, version 9.0.

## 3 Results

### 3.1 Clinical description

Six blood samples were collected from patients with sepsis infected by *K. pneumoniae*. The clinical characteristics of these cases are reported in Table 1. Most of the patients (4/6) were elderly, 4 out of 6 were women, and they were hospitalized in different department, except for cases Kpn2 and Kpn5 that were both admitted to the emergency department. The overall 30-day crude mortality rate was 50% (3/6), most of them (5/6) had diabetes and hypertension. Drainage catheters were applied in all six patients and they all with higher SOFA (Sequential Organ Failure Assessment) scores. When *K. pneumoniae* infections were diagnosed, all six patients had high fever, increased WBC count, elevated CRP and procalcitonin (PCT) levels.

**Table 1:** Clinical characteristics of the patients enrolled in the study.

Variables/patients	Kpn1	Kpn2	Kpn3	Kpn4	Kpn5	Kpn6
Clinical characteristics						
Age	86	89	69	91	80	62
Gender	Male	Female	Male	Female	Female	Female
Ward	Department of endocrinology	Emergency department	Respiratory department	Oncology department	Emergency department	ICU
Underlying conditions	Diabetes, hypertension	Diabetes, hypertension	COPD, pulmonary heart disease, hypertension, diabetes	Diabetes, hypertension, pneumonia	Diabetes, hypertension, pneumonia	Coronary heart disease, hypertension, acute severe pancreatitis
Empirical antimicrobial usage	Meropenem + teicoplanin + tigecycline	Meropenem + moxifloxacin	Piperacillin-tazobactam + etimicin + biapenem	Meropenem + moxifloxacin	Piperacillin-tazobactam + biapenem	Imipenem + tigecycline + sulbactam sodium + colistin
SOFA score	9	8	7	7	7	8
Clinical presentations						
Temperature (Tmax) (°C)	39.5	41	39.4	40	39.2	39
WBC (×109/L)	13.95	20.08	14.7	12.82	13.06	16.7
CRP (mg/dl)	11.3	19.3	41.2	20.4	10.9	15.9
PCT	9.65	15.96	8.33	13.94	16.59	12.09
Therapeutic antimicrobial usage	Amikacin + meropenem + linezolid + ceftazidime-avibactam	Vancomycin + tigecycline + aztreonam	Biapenem + ertapenem	Cefperazone-sulbactam	Biapenem + ertapenem	Ceftazidime avibactam sodium
Outcome (30 days)	Died	Alive	Alive	Died	Died	Alive

### 3.2 Antimicrobial susceptibility testing

Antimicrobial susceptibility results revealed that strains Kpn1, Kpn2, and Kpn6 were multidrug-resistant to *β*-lactams, carbapenems, aminoglycosides, fluroquinolones, sulfonamides, and tetracyclines. The exception was Kpn1, which was susceptible to amikacin and sulfamethoxazole. Kpn3 showed resistance to ceftazidime, ceftriaxone, cefepime, and an intermediate phenotype for levofloxacin. Interestingly, Kpn4 and Kpn5 were susceptible to all tested antimicrobial drugs (Table 2). Kpn1, Kpn2 and Kpn6 were identified as CRKp due to their resistance to carbapenems.

**Table 2:** susceptibility testing.

Antimicrobial drugs [MIC (mg/L)]	Kpn1	Kpn2	Kpn3	Kpn4	Kpn5	Kpn6
TZP	≥128.0(R)	≥128.0(R)	≤4(S)	≤4(S)	≤4(S)	≥128.0(R)
CAZ	≥64.0(R)	≥64.0(R)	32(R)	≤0.12(S)	≤0.12(S)	≥64.0(R)
CRO	≥64.0(R)	≥64.0(R)	≥64.0(R)	≤0.25(S)	≤0.25(S)	≥64.0(R)
FEP	≥64.0(R)	≥32.0(R)	≥32.0(R)	≤0.12(S)	≤0.12(S)	≥64.0(R)
CSL	≥64.0(R)	≥64.0(R)	≤8(S)	≤8(S)	≤8(S)	≥64.0(R)
ETP	≥8.0(R)	≥8.0(R)	≤0.12(S)	≤0.12(S)	≤0.12(S)	≥8.0(R)
IPM	≥16.0(R)	≥16.0(R)	≤0.25(S)	≤0.25(S)	≤0.25(S)	≥16.0(R)
AMK	≤2.0(S)	4(S)	≤2(S)	≤2(S)	≤2(S)	≥64.0(R)
LVX	≥8.0(R)	≥8.0(R)	1(I)	≤0.12(S)	≤0.12(S)	≥8.0(R)
SXT	≤20.0(S)	≥320.0(R)	40(S)	≤20(S)	≤20(S)	≥320.0(R)
TGC	≥16.0(R)	≥16.0(R)	2(S)	≤0.5(S)	1(S)	≥16.0(R)
ESBL	Neg	Pos	Pos	Neg	Neg	Neg

TZP = piperacillin-tazobactam; CAZ = ceftazidime; CRO = ceftriaxone; FEP = cefepime; CSL = cefperazone-sulbactam; ETP = ertapenem; IPM = imipenem; AMK = amikacin; LVX = levofloxacin; SXT = sulfamethoxazole; TGC = tigecycline; ESBL = extended spectrum beta-lactamase; Neg = negative; Pos = positive; R = Resistant; I = Intermediate; S = Sensitivity.

### 3.3 CPS and mucoviscosity measurement of six *K. pneumoniae* strains

In order to study the content of CPS, the uronic acid concentrations of 6 strains were quantitatively analyzed (Table 3). The CPS contents of Kpn1, Kpn2, Kpn3 and Kpn6 were significantly lower than Kpn4 and Kpn5. Next, mucoviscosity was assessed by determining the upper turbidity (optical density at 600 nm [OD600]) after low-velocity centrifugation. The mucoviscosity of Kpn1, Kpn2, Kpn3 and Kpn6 was significantly lower than Kpn4 and Kpn5. The uronic acid concentration and turbidity of Kpn4 and Kpn5 strains were not significantly different.

**Table 3:** CPS and mucoviscosity measurement of six *K. pneumoniae* strains.

Strain	CPS (nmol/mL)	Mucoviscosity (OD600)
Kpn1	84.14 ± 6.09*^#^	0.45 ± 0.05*^#^
Kpn2	90.60 ± 8.35*^#^	0.46 ± 0.04*^#^
Kpn3	76.13 ± 7.23*^#^	0.52 ± 0.06*^#^
Kpn4	121.08 ± 9.55	0.67 ± 0.09
Kpn5	136.92 ± 9.20	0.70 ± 0.08*^#^
Kpn6	83.46 ± 7.74*^#^	0.40 ± 0.03*^#^

Compared with Kpn4, the difference was statistically significant, *p* < 0.05*, Compared with Kpn5, the difference was statistically significant, *p* < 0.05^#^, CPS = capsular polysaccharide.

### 3.4 Phenotypes and molecular characterization

**Figure 1:**
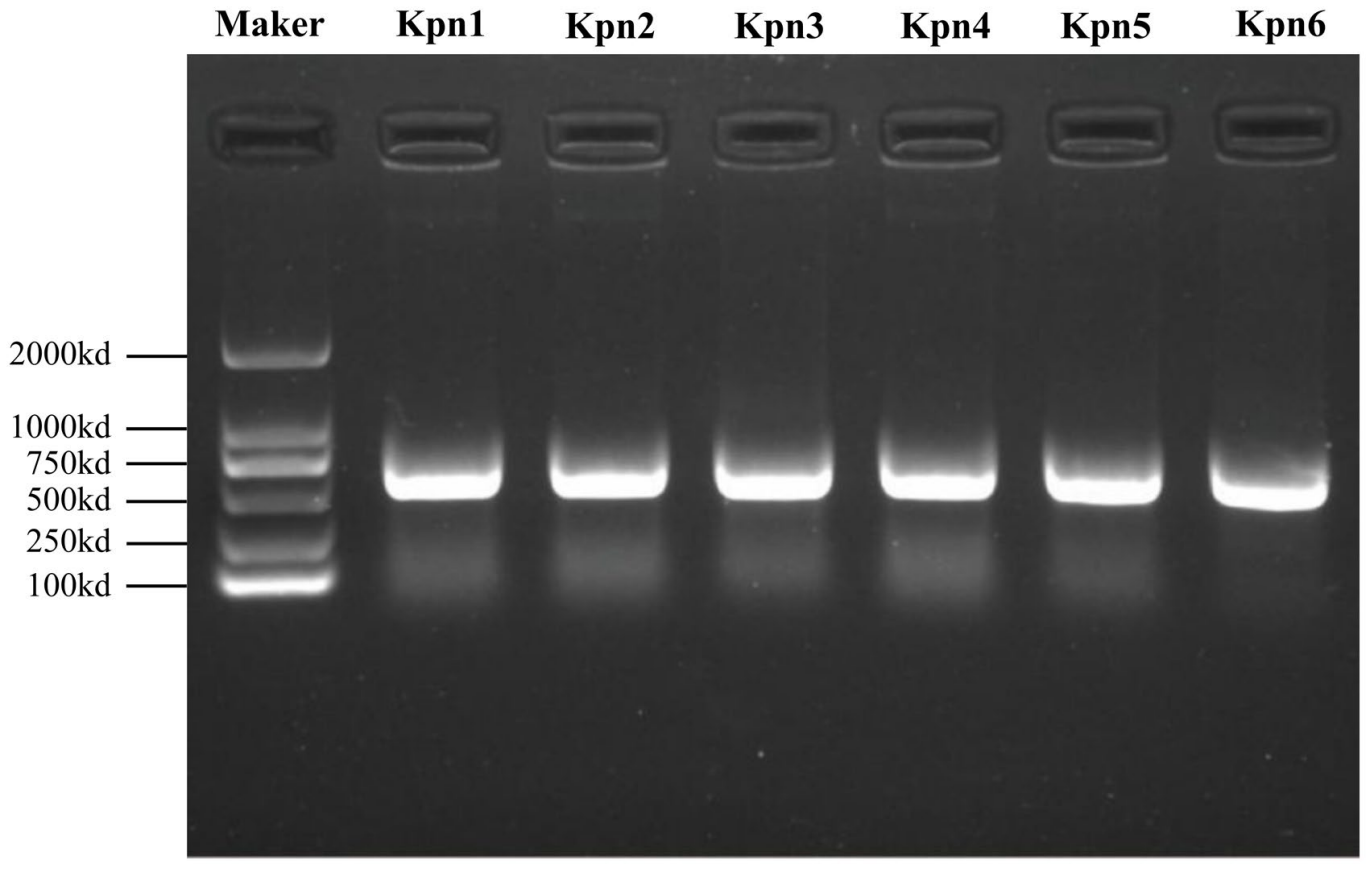
PCR amplification products of *wzi* gene of six strains with a size of 580-bp as compared with the DNA marker on the left side.

The *K. pneumoniae* infection in patients was confirmed by MALDI-TOF MS and Nanopore sequencing. The molecular typing showed that 6 strains belonged to 5 MLSTs (Supplementary Table 1). In detail, Kpn1 and Kpn6 were classified as ST11, Kpn2 and Kpn5 belonged to ST15 and ST23, while Kpn3 and Kpn4 belonged to ST950 and ST65, respectively. DNA was extracted for PCR amplification of 580 bp DNA fragments (Figure 1), followed by Sanger sequencing of *wzi* gene to identify the capsular (K) types. Simultaneously, we performed an analysis of K and lipopolysaccharide (O) loci using WGS data that provided results consistent with Sanger sequencing. Based on these analyses, Kpn1 and Kpn6 belonged to K64, Kpn2 to K19, Kpn5 to K1, and lastly Kpn3 and Kpn4 to K7 and K2, respectively (Supplementary Table 1). The lipopolysaccharide (O) types were described in Supplementary Table 1.

**Figure 2:**
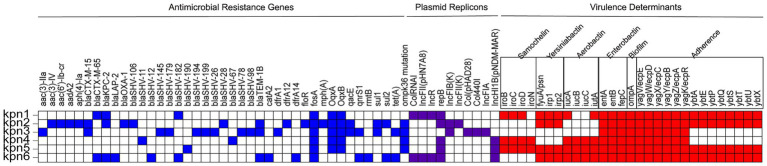
Genomic and AMR profiles of *K. pneumoniae* strains from this study. AMR genes, plasmid replicons, and virulence determinants are highlighted in blue, purple, and red, respectively. Blank squares indicate the absence of the genes.

Antimicrobial resistance genes, virulence determinants, and plasmid replicons are reported in Figure 2. Unexpectedly, all strains showed different kinds of multidrug resistance phenotypes mediated by different resistance mechanisms (Supplementary Table 1). Kpn1, Kpn2 and Kpn6 carried *bla*_KPC-2_ that is a resistance gene and resistant to carbapenems (Figure 2). Additionally, the strains carried multiple extended spectrum beta-lactamase (ESBL) genes, including *CTX-M*, *SHV*, *OXA-1*, *LAP-2* or *TEM* (Supplementary Table 1). Except Kpn4 and Kpn5 both with less drug resistance genes, other strains carried at least two or three beta-lactamases (Supplementary Table 1).

Porins are outer membrane proteins (OMPs), that usually aggregate to form pores, letting small hydrophilic molecules come across the membrane (Li et al., 2023). The *ompK35*, *ompK36*, and *ompK37* genes were detected in all 6 strains, and all of them reported point mutation in the *ompK37*, only Kpn2, Kpn3, Kpn4, and Kpn5 showed point mutations in *ompK36*. Point mutations in *ompK36* and *ompK37* can restrict the entry of *β* -lactam drugs into the bacteria and also limit the contact of β -lactam enzymes with drugs. It could lead to phenotypic sensitivity. While no mutations were found in *ompK35* in all six strains (Figure 2). Although no evidence of prior exposure to Fosfomycin was reported, all strains carried the associated *fosA* gene (Supplementary Table 1).

The VFs analysis showed that Kpn1 reported samochelin, yersiniabactin, aerobactin, enterobactin, biofilm, and adherence-related virulence genes, while knp2 did not encode genes for samochelin and aerobactin. For aerobactin, Kpn1 carried *iucA* and *iutA*, Kpn4, Kpn5 and Kpn6 carried *iucA*, *iucB*, *iucC*, and *iutA* (Figure 2). Kpn5 belonged to ST23-K1, which is the most common hypervirulent clone and reported had all 6 virulence genes including aerobactin. Kpn4, which belonged to the hypervirulent clone ST65-K2, carried salmochelin, aerobactin, enterobactin, biofilm virulence genes and the adherence-related virulence gene *yag*. Kpn3 carried enterobactin, biofilm and *yag*. Virulence determinants. Combined with mucoviscosity results, we confirm Kpn4 and Kpn5 to be hvKp.

Kpn1 and Kpn6 encoded at least four plasmid replicons, while Kpn4 and Kpn5 only harbored 2 plasmid replicons. These results suggested that hvKp has a lower number of plasmids than CRKp. Kpn1 and Kpn6 both contained plasmid replicons of ColRNAI, IncFII_(pHN7A8)_, IncR and repB, in addition, Kpn6 contained plasmid of IncHI1B_(pNDM-MAR)_. Kpn2 contained IncFIB_(K)_, IncFII_(K)_ and repB, while Kpn3 contained Col_(pHAD28)_, Col440I, IncFIA_(pBK30683)_, and IncFIB_(K)_. Kpn5, similarly to Kpn4, contained 2 replicons, repB and IncHI1B_(pNDM-MAR)_ (Table 4).

**Table 4:** Detection of plasmid and related characteristic of six *K. pneumoniae* isolates.

Isolates	Number of plasmids	Plasmid replicons	KPC-2
Kpn1	5	ColRNAI-1, ColRNAI-2, IncFII_(pHN7A8)_, IncR, repB	Yes
Kpn2	7	IncFIB_(K)_, IncFII_(K)_, repB	Yes
Kpn3	5	Col_(pHAD28)_, Col440I-1, Col440I-2, IncFIA_(pBK30683)_, IncFIB_(K)_	No
Kpn4	2	IncHI1B_(pNDM-MAR)_, repB	No
Kpn5	2	IncHI1B_(pNDM-MAR)_, repB	No
Kpn6	7	ColRNAI-1, ColRNAI-2, IncFII_(pHN7A8)_, IncHI1B_(pNDM-MAR)_, IncR, repB	Yes

These results suggested a significant diversity of strains isolated from blood.

### 3.5 Phylogenetic analysis

**Figure 3:**
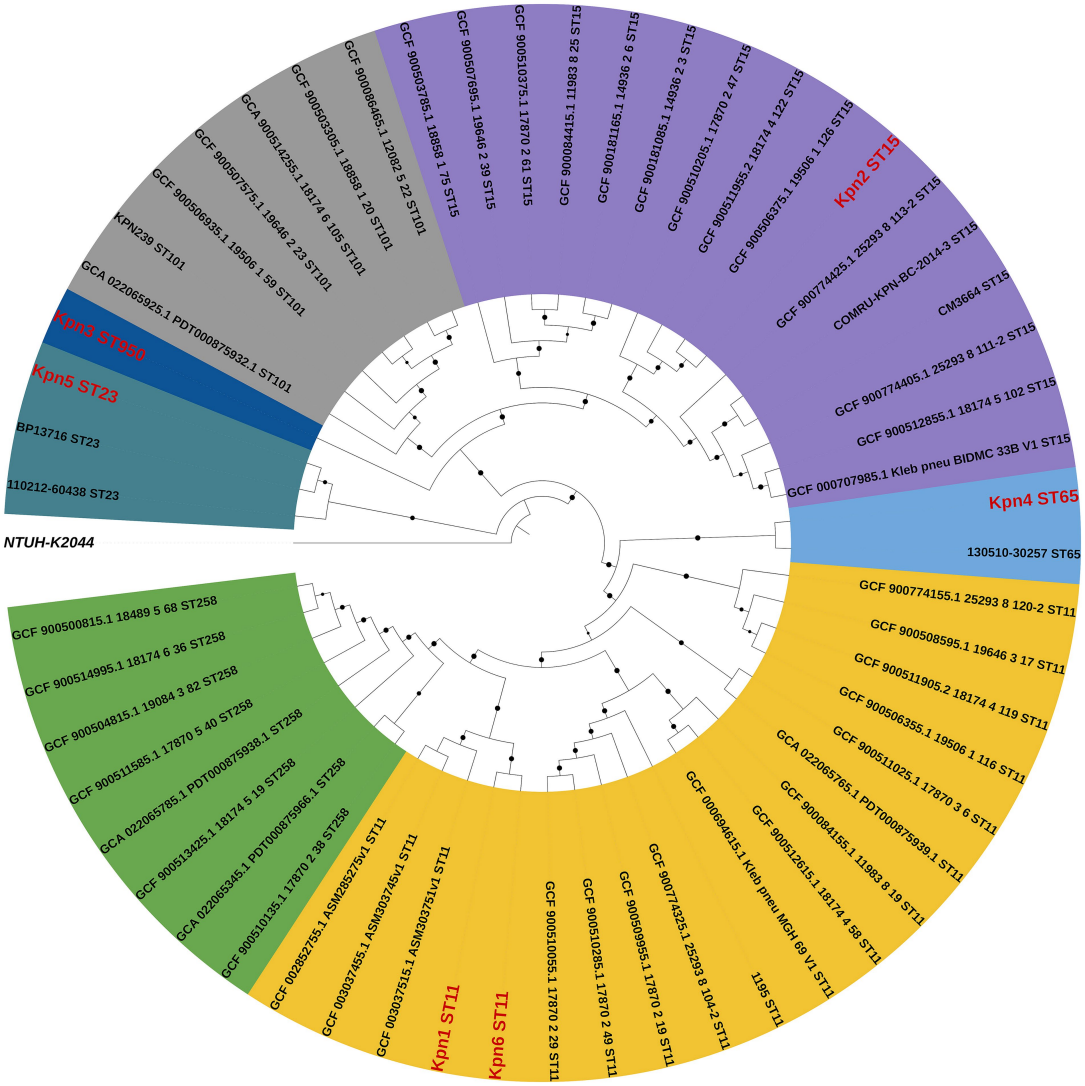
Comparative phylogenetic analysis of six *K. pneumoniae* isolates from this study (red) and reference strains. The percentages of replicate trees in which the associated taxa clustered together based on a 1,000 replicates-based bootstrap test are reported as black dots at the branches level.

Phylogenetic analysis included four strains (ST11, ST15, ST101, and ST258) widely studied in Europe, as well as two highly virulent clones of ST23 and ST65 (Wyres KA-O et al., 2020). The phylogenetic analysis showed that all isolates clustered in two subclades, and the 6 strains from this study belonged to 5 lineages (Figure 3). Kpn1 and kpn6 were closely related to Eastern Asia, suggesting they originated from Eastern Asia. Additionally, Kpn4 was closely related to Southeastern Asia, while Kpn2 was more closed to the strains from North Europe, suggesting their origins as well. Kpn3 belonged to ST950, which is closely related to Kpn2, while Kpn4 was closely related to Kpn1 and Kpn6. In addition, the phylogenetic tree showed that strains from this study belonged to two different subclades: the first included Kpn1, Kpn2, Kpn3, Kpn4, and Kpn6, while Kpn5 clustered alone in another subclade.

### 3.6 Comparison of plasmid profiles of CRKp isolates

**Figure 4:**
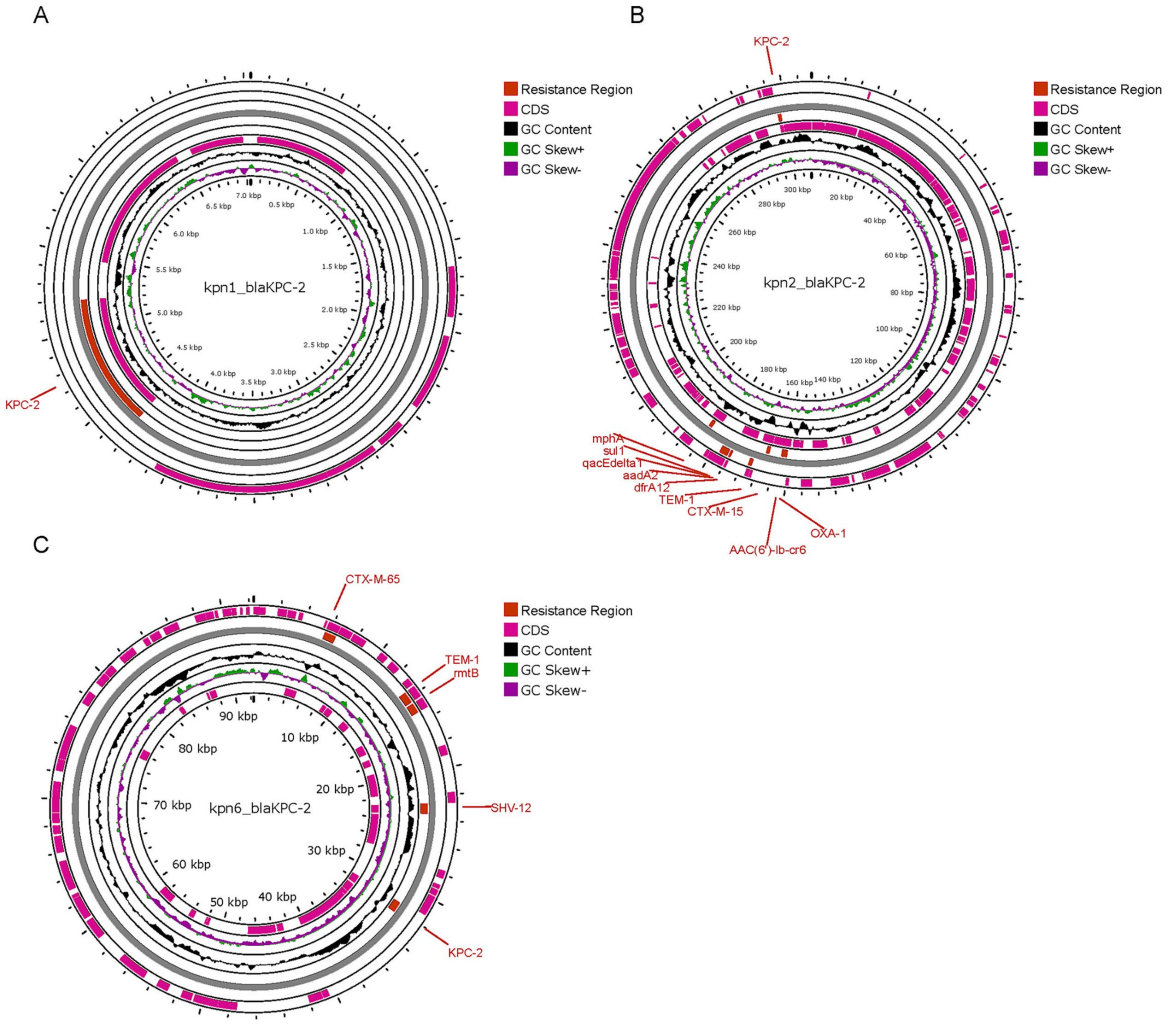
Comparison between bla_KPC-2_ plasmid of 3 CRKp isolates. **(A)** plasmid of Kpn1, designated as Kpn1_bla_KPC-2_; **(B)** plasmid of Kpn2, designated as Kpn2_bla_KPC-2_; **(C)** plasmid of Kpn6, designated as Kpn6_bla_KPC-2_.

The six isolates all contained plasmid replicons: Kpn4 and Kpn5 contained 2 plasmid replicons, while the other strains contained 5 or 7. A plasmid analysis was performed on Kpn1, Kpn2 and Kpn6, which were CRKp isolates. The gene responsible for carbapenem-resistance (*bla*_KPC-2_) was identified in plasmids contained in Kpn1, Kpn2, and Kpn6, respectively. Kpn1 and Kpn6 belonged to the same MLST type, but the plasmid carrying *bla*_KPC-2_ was different. In detail, the plasmid of Kpn1 was 7.04 kb in length and carried only *bla*_KPC-2_. The plasmid of Kpn6 was 92.3 kb in length, and it carried *bla*_KPC-2_ as well as other *β*-lactamase-encoding genes such as *bla*_*C*TX-M-65_, *bla*_TEM-1_, and *bla*_SHV-12_, besides the resistance gene *rmtB* and a plasmid replicon of IncFII/IncR. Kpn2 belonged to ST15, and its plasmid of 307.31 kb in length carrying *bla*_KPC-2_. The plasmid of Kpn2 encoded for three β-lactamase-encoding genes (*bla*_CTX-M-15_, *bla*_TEM-1B_, and *bla*_OXA-1_). Several additional antimicrobial resistance genes were also detected such as *dfrA12*, *mph(A)*, *sul1*, *aadA2*, *qacE,* and *AAC (6′)-lb-cr6* (Figure 4), along with plasmid replicons of IncFII, repB, and IncFIB. More significantly, in Kpn1, there was also a plasmid carrying VFs as *iutA*, *iucA*, *iroD*, *iroC*, and *iroB* along with the antimicrobial resistance gene of *bla*_CTX-M-65_ and with a repB plasmid. No VFs plasmid carrying antimicrobial resistance gene was identified in Kpn6. These results indicated that although Kpn1, Kpn2, Kpn6 all belonged to CRKp, *bla*_KPC-2_ gene location was different and there was obvious diversity among those bla*_KPC-2_* carrying plasmid.

### 3.7 Survival probability analysis during blood infection in mice

**Figure 5:**
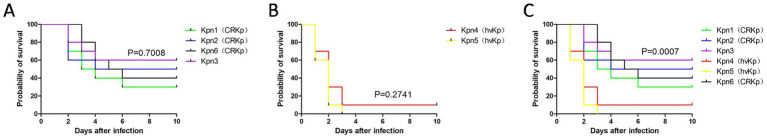
Survival curves after infection among *K. pneumoniae* isolates. **(A)** Survival curve of Kpn1, Kpn2, Kpn3, Kpn6; **(B)** survival curve of Kpn4 and Kpn5; **(C)** survival curve of six *K. pneumoniae* strains.

To test the virulence of different strains. We infected mice treated with cyclophosphamide and cortisone via a tail vein with 6 strains isolated from patient blood. As measured by mortality, mice infected with Kpn1, Kpn2, Kpn3, Kpn6 had no statistical difference in mortality between the groups (Figure 5A). Mice infected with Kpn4 and Kpn5 also had no statistical difference in mortality between the groups (Figure 5B). However, the death probability of the six strains together showed a statistical difference, indicating that hvKp is more virulent and can cause higher mortality (Figure 5C). This result is consistent with what we have seen in patients.

## 4 Discussion

*K. pneumoniae* has become one of the major pathogens involved in hospital infections (Xie et al., 2021). *K. pneumoniae* infection may cause pneumonia, urinary tract infections (UTIs), and bloodstream infections (Martin and Bachman, 2018). In particular, the bloodstream infections caused by *K. pneumoniae*, which is called bacteremia can lead to a severe sepsis (Holmes et al., 2021). In our study, 6 clinical cases with bloodstream infections caused by *K. pneumoniae* were analyzed. More vulnerable patients like neonates, elderly, or the immunocompromised ones with inserted medical devices are more susceptible to bacteria (Wyres KA-O et al., 2020; Hu et al., 2021). Diabetes is a predisposing factor for *K. pneumoniae-*related severe community-acquired infections (CAI) (Wyres KA-O et al., 2020). Our study also confirmed that elderly patients with hypertension or diabetes are at high risk for *K. pneumoniae* bloodstream infection. There was no difference between the sexes of the infected people and they all had a high inflammatory response.

CRKp are the carbapenem-resistant *K. pneumoniae* strains while hvKp are the hypervirulent strains. Kpn1, Kpn2, and Kpn6 were CRKp strains based on the presence of related genes and phenotype. Kpn4 and Kpn5 were identified as hvKp strains (Choby et al., 2020; Wyres KA-O et al., 2020; Sohrabi et al., 2022). Our results show that the mortality rate of mice with hvKp is higher than that with CRKp. We guess that the higher mortality rate may be due to the higher CPS content and mucoviscosity. hvKp was commonly susceptible to antimicrobials (Wyres KA-O et al., 2020). In this study, both Kpn4 and Kpn5 were both susceptible to all tested drugs. The results of drug sensitivity indicated that high virulence and multiple drug resistance generally did not exist in one strain at the same time. Kpn3 belonged to ST950, which is a rarely detected type, and further research is needed to determine its genetic and phenotypic characteristics.

In our study, Kpn1, Kpn2 and Kpn6 were CRKp strains and resistant to almost all tested drugs, however, they did not belong to the same serotype. Kpn1 and Kpn6 both belonged to ST11 and their K types were K64 and O types were O2a. Kpn2 belonged to ST15, and K19 and O1 types. *K. pneumoniae* strains are intrinsically resistant to ampicillin, while their resistance to other drugs is acquired through chromosomal mutations and large conjugative plasmids (Wyres and Holt, 2018). The mechanism of resistance for carbapenem of ST11 CRKp was mainly based on the presence of carbapenemase such as *bla_KPC-2_*, *bla_NDM-1_* and *bla_OXA-48_*. Broad-spectrum *β*-lactam resistance genes in ST11 CRKp included *bla_CTX-M_*, *bla_SHV_* and *bla_TEM_* (Liao et al., 2020). In this study, Kpn1 and Kpn6 were both resistant to broad-spectrum β-lactam (including carbapenem) carrying *bla*_KPC-2_ and extended-spectrum β-lactamase (ESBL) genes. It is worth noting that they carry different ESBL genes, that were *bla_SHV-182_* and *bla_CTX-M-65_* for Kpn1, while were *bla_SHV-12_*, *bla_SHV-182_*, *bla_CTX-M-65_*, *bla_TEM-1B_* and *bla_LAP-2_* for Kpn6. The results show that although Kpn1 and Kpn6 belong to ST11and had the same resistance phenotype, their resistance genes are not the same. So, they have different resistance mechanisms and antibiotic sensitivity. These may lead to a different therapeutic effect. In our study, Kpn2 belonged to ST15, and K19 and O1 types. The isolate carried the carbapenemases-encoding genes *bla_KPC − 2_*, the β-lactamases-encoding genes *bla_TEM-1B_*, *bla_SHV-106_*, *bla_SHV-28_*, *bla_OXA-1_* and *bla_CTX − M − 15_*. Kpn2 was CRKp, just like Kpn1 and Kpn6. However, the drug resistance genes carried by Kpn2 are different from those of Kpn1 and Kpn6. The results show that different drug resistance genes will lead to the same CRKp phenotype. Kpn4 and Kpn5 were susceptible to all tested antimicrobial drugs, despite the detection of some resistance genes such as the β-lactamases. Notably, Kpn3 was a relatively new clone in China, it carried the β-lactamases-encoding genes *bla_TEM-1B_*, *bla_CTX − M − 15_*, *bla_SHV-98_*, *bla_SHV-78_*, *bla_SHV-26_*, *bla_SHV-194_*, *bla_SHV-199_*, *bla_SHV-179_* and *bla_SHV-145_*. Kpn3 did not belong to CRKp and had the characteristics of resistance to ceftazidime and cefepime and intermediate resistance to levofloxacin. It is different from CRKp strains, which are extensively resistant, meanwhile unlike hvKp strains with the characteristics of sensitive to all tested drugs. Further research is needed to determine its genetic characterization.

HvKp strains are emerging pathogens responsible for community and hospital-acquired infections with a high rate (Choby et al., 2020). Several accessory gene-encoded virulence factors were detected in *K. pneumoniae*, including four siderophore systems, capsule, K1 and K2 capsule types, colibactin toxin (Russo and Marr, 2019), and aerobactin which is considered a significant virulence factor of hvKp strains (Wang et al., 2020). Above all, hypermucoidy is likely the most well-known virulence determinant for *K. pneumoniae* (Wyres KA-O et al., 2020; Yang et al., 2022). The genetic factors of hypervirulence are often encoded on large virulence plasmids or chromosomal mobile genetic elements (Wang et al., 2020). Siderophore system was identified as the primary mechanism of iron acquisition in *K. pneumoniae*. Salmochelin and aerobactin are hvKp specific (Russo and Marr, 2019). In our study, Kpn4 and Kpn5 were hvKp, and they contained both salmochelin and aerobactin. And similar to the standard strain of hvKp NUTH-K2044, Kpn4 and Kpn5 both harbor plasmids of IncHI1B (pNDM-MAR) and repB. Besides, our mice survival experiments have demonstrated their characteristic of hypervirulent. Surprisingly, Kpn1 contained salmochelin of *iroB*, *iroC*, *iroD* and contained aerobactin of *iucA* and *iutA*, Kpn6 contained 4 kinds of aerobactin. Considering that Kpn1 and Kpn6 are CRKp strains, they have acquired some characteristics of hvKp. However, another CRKp strain of Kpn2 did not get the characteristics associated with hvKp.

MDR *K. pneumoniae* carried more plasmid replication types than hvKp (Du et al., 2022). Indeed, in our study, Kpn1, Kpn2 and Kpn6 were MDR strains, carrying more plasmid replication types when compared to Kpn4 and Kpn5. IncF plasmids were associated with a higher transfer rate of different antibiotic resistance genes (Kopotsa et al., 2019). The *bla*_KPC-2_-bearing plasmid is preferentially located in the IncFII plasmid (Gao et al., 2020). And our data showed that the *bla*_KPC-2_ gene was located on the IncFII plasmid in Kpn2 and Kpn6, contrarily to Kpn1. The results showed the diversity of *bla*_KPC-2_-bearing plasmid. This difference may be responsible for the susceptibility of Kpn1 to AMK and SXT.

The phylogenic analysis revealed that Kpn1 and Kpn6 (both ST11-K64) originated from Eastern Asia and their lineages were closely related, and also being the hospitalization of patient Kpn1 and Kpn6 overlapped, suggesting a potential local transmission lineage. However, given the differences of their antimicrobial resistance, virulence determinants, and *bla*_KPC-2_ -bearing plasmid profiles (*bla*_KPC-2_ co-located with *bla*_CTX-M-65_ in Kpn6 but not in Kpn1), it is likely that *K. pneumoniae* evolved while spreading. These results highlight the plasticity of accessory genomes in ST11 clones during hospital spread. ST11 was described as the predominant CRKp clone in China (Jin et al., 2021), and Kpn1 and Kpn6 were found to belong to ST11, indicating the need to increase its surveillance. Kpn2 (ST15-K19) exhibited phylogenetic proximity to Northern European strains, possibly indicating an imported lineage. This aligns with reports of ST15 CRKp dissemination in Europe via plasmids like IncFII (Martins et al., 2020). Furthermore, Kpn4 and Kpn5 were belonged to hvKp and not grouped into the same lineage. Kpn4 (ST65-K2) showed genetic similarity to Southeast Asian isolates, supporting the hypothesis that hvKp clones may spread regionally through travel or trade (Wyres KA-O et al., 2020). Kpn5 (ST23-K1) was a globally prevalent hvKp clone and clustered separately from other isolates. Consistent with its distinct virulence plasmid profile (IncHI1B/repB) and community-associated epidemiology (Wang et al., 2020; Zhou et al., 2023). Kpn3 (ST950-K7) occupied an intermediate phylogenetic position between CRKp and hvKp clades. Its genetic distance from other strains underscores the need to monitor novel sequence types for hybrid virulence-resistance traits. The divergence between Kpn1/Kpn6 (ST11) and Kpn2 (ST15) implies that carbapenem resistance in this setting arose through both local evolution (*bla*_KPC-2_ plasmid acquisition in ST11) and external introductions (ST15). Overall, the 6 strains belonged to 5 lineages, indicating that the colony sources of *K. pneumoniae* bloodstream infection were diverse. This could also be due to the international environment of Beijing as a metropolis, with people from all over the world, which may have caused the spread of different source of *K. pneumoniae*.

To study the diversity of the KPC-2 gene, we compared *bla*_KPC-2_ -bearing plasmid of three CRKp clones and we found that they were of different sizes. Several studies reported a co-location of *bla_KPC-2_* and *bla_CTX-M-65_* in *K. pneumoniae* (Chen et al., 2022). In this study, we found that the *bla_KPC-2_* and *bla_CTX-M-65_* genes were located on a ~ 92.3 kb IncFII/IncR plasmid in Kpn6. This co-localization with aminoglycoside resistance genes likely facilitated horizontal transfer of multidrug resistance. Notably, both Kpn1 and Kpn6 belonged to the same serotype ST11, however, *bla_KPC-2_* and *bla_CTX-M-65_* genes in Kpn1 were not located on the same plasmid, and neither of them was located on the IncFII/IncR plasmid. This segregation may explain Kpn1’s retained susceptibility to amikacin and sulfamethoxazole, as the resistance genes were not amplified on a single high-copy plasmid. We speculate that this reason leads to the difference in antibiotic resistance between kpn1 and kpn6. ESBLs, carbapenemases, and aminoglycoside AMR genes on the same plasmid in Kpn6. ESBLs, carbapenemase, quinolone, trimethoprim, streptomycin, and aminoglycoside resistance genes were co-located on a ~ 307.31 kb IncFII/IncFIB plasmid in Kpn2. This result proved that the KPC-2 plasmid carries different resistance genes in different strains. The co-existence of multiple AMR genes on the same plasmid limited the choices of antimicrobials, which has become a critical problem for clinical therapy. Notably, *bla_CTX-M-65_* gene and virulence factors *iucA~C* and *iutA* were co-located on the same plasmid in Kpn1. As for the patient that was infected by Kpn1, his death may be attributed to the severe conditions caused by the existence of a hybrid plasmid carrying AMR genes and virulence-encoding genes. These results confirmed the diversity of plasmids carrying the KPC-2 gene. The exact relationship between plasmids and drug resistance needs to be confirmed through coupling tests or sequencing resolution analysis.

Despite important clinical implications, this study has still some limitations. This study only included six isolates of *K. pneumoniae* from one hospital, which limited the universality of the research results. It can only represent the genomic diversity of CRKp and hvKp strains in the blood of patients in a single center. Although phylogenetic analysis indicated the potential geographical origin of the strain, no detailed patient contact tracing or environmental sampling was conducted. Therefore, it is impossible to confirm the direct transmission event. Although WGS identified resistance and virulence genes, phenotypic assays (such as plasmid conjugation experiments) were not conducted. Functional studies are needed to confirm the biological impact of genetic variations (such as ompK36 mutations or hybrid plasmids). These limitations highlight the need for larger-scale, functionally validated studies to translate genomic discoveries into feasible clinical or public health interventions.

## 5 Conclusion

In summary, we found that *K. pneumoniae* bloodstream infection strains have diversity in drug resistance genes, virulence factors, plasmids, etc. These strains have both CRKp and hvKp. The diversity reminds us that we should pay attention to genomic information in the treatment process, hoping to provide new ideas for personalized medicine.

## Data Availability

The raw sequence data reported in this paper have been deposited in the Genome Sequence Archive in National Genomics Data Center, China National Center for Bioinformation/Beijing Institute of Genomics, Chinese Academy of Sciences (GSA: CRA026949) that are publicly accessible at: https://ngdc.cncb.ac.cn/gsa.

## Footnotes

^1^ https://www.eucast.org/clinical_breakpoints

^2^ http://www.genomicepidemiology.org/services/

^3^ http://www.mgc.ac.cn/VFs/

^4^ https://github.com/katholt/Kaptive

^5^ https://proksee.ca
